# Elephant Cathelicidin-Derived Peptides Inhibit Herpes Simplex Virus 1 Infection

**DOI:** 10.3390/antibiotics14070655

**Published:** 2025-06-28

**Authors:** Haiche Yisihaer, Peng Dong, Pengpeng Li, Enjie Deng, Rui Meng, Lin Jin, Guilan Li

**Affiliations:** Shanxi Key Laboratory for Modernization of TCVM, College of Veterinary Medicine, Shanxi Agricultural University, Jinzhong 030801, China; s20222385@stu.sxau.edu.cn (H.Y.); s20222404@stu.sxau.edu.cn (P.D.); ppli@aptbiotech.com (P.L.); 20232420@stu.sxau.edu.cn (E.D.); 202430919@stu.sxau.edu.cn (R.M.)

**Keywords:** HSV-1, *Elephas maximus*, cathelicidin, peptide modification, antiviral

## Abstract

Herpes simplex virus type 1 (HSV-1) is a globally prevalent pathogen that can infect a variety of animal species as well as humans. However, existing antiviral therapies are constrained in their capacity to effectively target viral latency and prevent recurrent infections. Antimicrobial peptides (AMPs), particularly cathelicidins, as part of innate immune system have demonstrated broad-spectrum efficacy against viral pathogens. In this study, four peptides derived from *Elephas maximus* cathelicidin EM were designed and optimized (EM-1 to EM-4). We identified low toxicity peptide derivatives through hemolytic and cytotoxicity assays, quantified their anti-HSV-1 activity by determining IC_50_. Antiviral mechanisms were investigated using RT-qPCR and antiviral efficacy was ultimately validated in C57BL/6J mice through viral load quantification in brain, lung, and heart tissues. Our findings revealed that EM-1 significantly inhibited HSV-1 replication in U251 cells. In a murine footpad inoculation model, EM-1 administration substantially reduced viral loads and alleviated inflammatory responses. Histological assessment demonstrated that EM-1 treatment mitigated HSV-1 induced tissue damage in infected mice. We also found that EM-1 exerted its antiviral effects by upregulating the expression of interferon-gamma and its downstream genes, such as *ISG15* and *MX1*. These findings indicated that EM-1 is a dual function peptide that inhibits replication of HSV-1 as well as enhances host antiviral immunity. Collectively, this study highlights the therapeutic potential of elephant cathelicidin derived peptides in antiviral development.

## 1. Introduction

Herpes simplex virus type 1 (HSV-1) is an enveloped virus containing a double-stranded DNA genome and belongs to the *Herpesviridae* family. As a widely prevalent pathogen, HSV-1 presents multifaceted public health challenges. According to the World Health Organization (WHO), HSV-1 infection affects over 3.7 billion people globally under the age of 50. HSV-1 can breach the blood brain barrier, causing herpes simplex encephalitis (HSE). It is also a leading cause of infectious blindness in developed nations. These severe symptoms pose significant threats to both neurological and visual health [1]. HSV-1 further contributes to orofacial pathology, including herpes labialis [2]. Following initial infection, the virus establishes lifelong latency in trigeminal ganglia neurons. Periodic reactivation enables renewed viral replication, driving recurrent clinical symptoms [3]. Acyclovir (ACV) is the main treatment for HSV-1 infections. Viral thymidine kinase (TK) and host cell kinases sequentially phosphorylate ACV, converting ACV into its active triphosphate derivative [4,5,6]. This activated form inhibits viral DNA polymerase, thereby blocking viral replication. However, the extensive clinical use of ACV has driven the emergence of drug resistance. Considering the significant threats that HSV-1 poses to public health, it is essential to discover novel molecules with potent anti-HSV-1 effects. Furthermore, elucidating their mechanisms of action is also crucial.

Over the past two decades, antimicrobial peptides (AMPs) have emerged as promising antibiotic alternatives with antiviral activity. As of January 2025, 5099 AMPs have been reported in the Antimicrobial Peptide Database (APD; https://aps.unmc.edu). AMPs are present in the innate immune system of almost every living organism, including both invertebrates and vertebrates. Among these AMPs, the cathelicidin family is a key component of the innate immune system, being widely distributed across mammals, birds, reptiles, and fish [7]. Cathelicidins exhibit direct antimicrobial activity as well as immunomodulatory effects [8,9]. Human cathelicidin LL-37 can inhibit HSV-1 infection by disrupting the viral envelope and it also effectively suppress infections caused by HIV, Vaccinia virus, and Influenza virus [10,11,12]. Cathelicidin BF-30 inhibits Influenza A virus infection by causing virion membrane fusion [13]. Moreover, cathelicidins also interfere with host pathogen interactions. For example, cyclic β-sheet θ-defensins impede HSV-1 adhesion and entry by binding to the gB protein [14]. Venom peptides Hp1036 and Hp1239 inactivate HSV-1 particles and suppress infection by blocking viral adsorption and cellular invasion [15]. Cathelicidins modulate the immune response by regulating the expression of pro-inflammatory cytokines and their receptors. A typical example is bovine BMAP28, which exerts dual regulatory effects in murine RAW264.7 macrophages by enhancing TNF-α secretion while simultaneously suppressing TLR4 activation and receptor endocytosis. This coordinated action disrupts the TLR4 signaling cascade, impeding the activation of downstream signaling molecules and the transcription factor IRF3, thereby attenuating type I interferon production through reduced IFN-β expression [16]. These studies indicate that antimicrobial peptides combat viruses by targeting key steps in the viral life cycle. Importantly, this functionality distinguishes them from conventional antibiotics and contributes to their significantly lower tendency to induce microbial resistance [17]. However, AMPs face challenges such as enzymatic susceptibility and poor bioavailability. Current strategies focus on optimizing peptide length and charge distribution to enhance stability and potency. For example, truncated peptides ZN-5 and ZN-6, designed from Oreoch-2, showed improved antimicrobial activity and reduced hemolysis [18]. Cationic residue substitution (Arg/Lys) also boost efficacy by increasing positive charge density, as seen in HJH-2, HJH-3 [19], abhisin [20,21], abaecin [22], RN15 [23], and IL15 [24]. Collectively, cathelicidin research holds promise for developing new antimicrobial agents to address growing infection threats.

In this work, we evaluated the antiviral activity of Asian elephant cathelicidin. Specifically, we examined the antiviral activity and underlying mechanisms of Asian elephant cathelicidin and its derived peptides against the HSV-1. Our findings may offer an important strategy for the development of novel antiviral drugs.

## 2. Results

### 2.1. Design of Cathelicidin EM and Its Derivative Peptides

**Figure 1:**
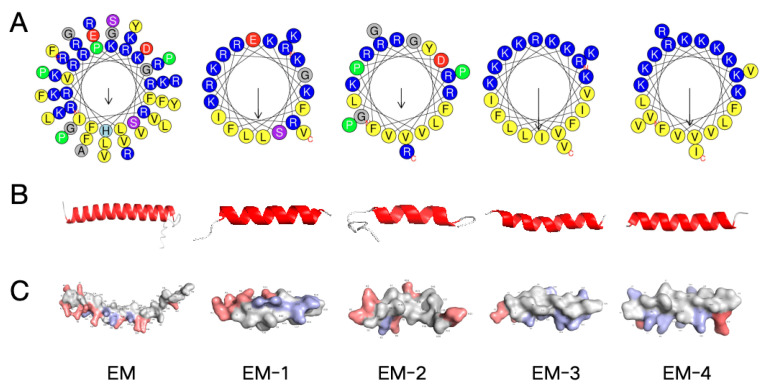
The amphipathic helix structure and three dimensional structure projections of cathelicidin EM and derivative peptides. (**A**) The Shiffer Edmundson wheel projection of the template peptide EM and its modified peptides using HeliQuest analysis (https://heliquest.ipmc.cnrs.fr/index.html, accessed on 26 December 2022); the C-terminus and N-terminus are indicated by “C” and “N”. Residues are color-coded with non-polar hydrophobic residues in yellow, polar basic residues in dark blue, negatively charged residues in red, hydrophilic residues in purple, histidine in light blue, glycine in gray, and proline in green circles. (**B**) Three dimensional structure projections of the template peptide EM and its modified peptides using I-TASSER (https://zhanggroup.org/I-TASSER/, accessed on 26 December 2022); the different colors represent various secondary structure types, with helix in red and coil in white. (**C**) Potential surfaces of peptides.

To identify the template for antiviral peptides, we conducted a search in NCBI (https://www.ncbi.nlm.nih.gov/, accessed on 20 December 2022). The cathelicidin mature peptide from *Elephas maximus* was identified and named EM as the template. Through sequence optimization, four modified peptides EM-1, EM-2, EM-3, and EM-4 were designed. The modification principles are as follows: the proportion of polar residues and non-polar residues is about 50%; the distribution of positively charged amino acids is relatively concentrated; and hydrophobic amino acids are embedded on the hydrophilic side of the helical structure. The Schiffer Edmundson helical wheel projections of the template peptide EM and its modified peptides are shown in Figure 1A, while the three dimensional structure projections and potential surfaces of all peptides are displayed in Figure 1B,C.

### 2.2. Safety Evaluation of Cathelicidin EM and Its Derivative Peptides

**Figure 2:**
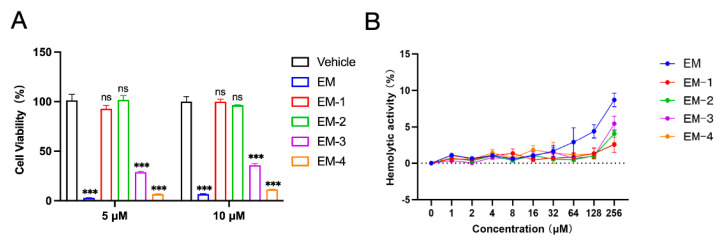
Cytotoxicity and hemolytic activity of cathelicidin EM and its derived peptides. (**A**) Cytotoxicity analysis of cathelicidin EM and its modified peptides on U251 cells at different concentrations. All statistical significance refers to comparisons with the vehicle group. (**B**) Hemolytic analysis of cathelicidin EM and its modified peptides on rabbit erythrocytes at different concentrations. Maximum hemolysis was determined by adding 0.1% Triton X-100 to a sample of cells. Hemolysis of testing samples was calculated as the percentage of Triton X-100. The data represent three independent experiments and are presented as the mean ± SEM. Statistical significance was determined by a two-tailed Student‘s *t*-test. *** *p* < 0.001; ns, not significant.

Safety evaluation of all peptides were conducted through hemolytic and cytotoxicity assays. The cytotoxic effects of cathelicidin EM and its derivative peptides on U251 cells was evaluated using the CCK-8 assay at concentrations of 5 μM and 10 μM. Results revealed that the modified peptides EM-1 and EM-2 exhibited significantly lower toxicity compared to peptide EM, EM-3, and EM-4 (Figure 2A). Hemolysis assays were performed using rabbit erythrocytes in liquid culture medium. Results demonstrated that all modified peptides showed significantly improved hemocompatibility compared to the peptide EM (Figure 2B). Both hemolytic and cytotoxicity results indicated that EM-1 and EM-2 exhibited notable safety advantages. Therefore, EM-1 and EM-2 were selected for further investigation.

### 2.3. The Peptide EM-1 Inhibits HSV-1 Infection In Vitro

**Figure 3:**
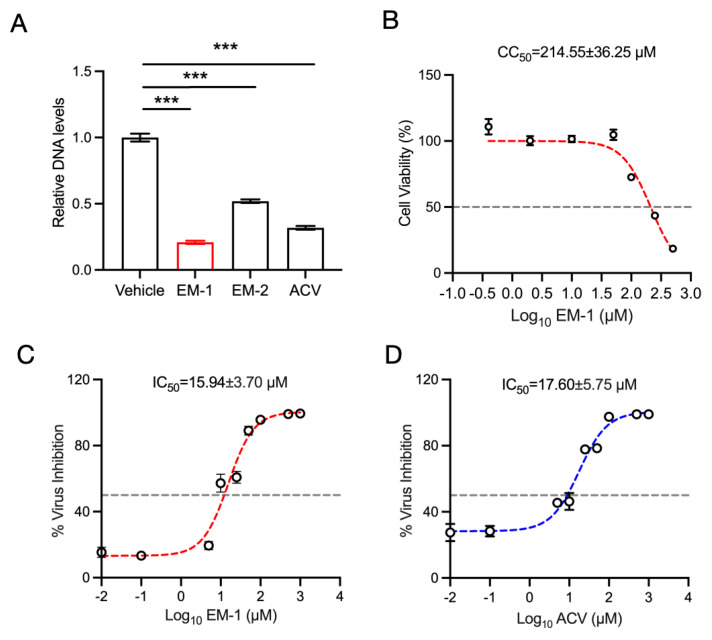
Peptide EM-1 exhibits antiviral activity against HSV-1 in vitro. (**A**) qPCR analysis of relative DNA levels of HSV-1 *UL30* in U251 cells. Cells were infected with HSV-1 (MOI = 0.1) for 24 h and treated with 10 μM EM-1, EM-2, or the positive control acyclovir (ACV). Viral DNA levels were quantified by qPCR targeting the *UL30*. Results are presented relative to the reference gene 18s and vehicle group. (**B**) U251 cells were treated with different concentrations of EM-1 and cell viability was detected CC_50_ after 24 h by using CCK-8 assay. (**C**) The IC_50_ of EM-1 on the gene of HSV-1. (**D**) The IC_50_ of ACV on the gene of HSV-1. The data represent three independent experiments and are presented as the mean ± SEM. Statistical significance was determined by a two-tailed Student‘s *t*-test. *** *p* < 0.001.

Firstly, we evaluated the anti-HSV-1 activity of EM-1 and EM-2 in vitro. U251 cells were infected with HSV-1 (MOI = 0.1), followed by co-treatment with EM-1 and EM-2. Total DNA was extracted and subjected to qPCR for detection of DNA levels. As shown in Figure 3A, both peptides EM-1 and EM-2 exhibited marked suppression of HSV-1 activity compared to the virus control group (*p* < 0.001), exhibiting superior antiviral efficacy. Based on this, cell viability was determined by the CCK-8 assay to identify the median cytotoxic concentration (CC_50_) of EM-1 (Figure 3B). To further investigate the antiviral activity of EM-1, we plotted a dose response curve to determine the half-maximal inhibitory concentration (IC_50_). The results (Figure 3C,D) demonstrated that the IC_50_ of EM-1 against HSV-1 was 15.94 ± 3.70 µM, while that of the positive drug acyclovir (ACV) was 17.60 ± 5.75 µM, supporting its potential as a promising alternative treatment. Therefore, EM-1 was selected for further exploration of its antiviral mechanisms.

### 2.4. The Peptide EM-1 Induces Type Ⅱ Interferon and Its Downstream Gene Expression

**Figure 4:**
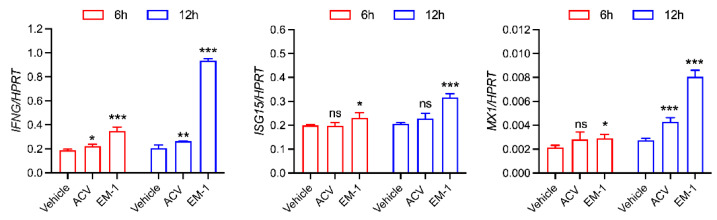
EM-1 upregulates the expression of interferon-gamma and its downstream gene. Total RNA was extracted and subjected to RT-qPCR for detection of mRNA levels. RT-qPCR analysis of *IFNG* and its downstream genes *ISG15* and *MX1* in U251 cells at 6 h or 12 h with 10 µM EM-1 and 10 µM ACV. The expression levels of *IFNG*, *ISG15*, and *MX1* are presented relative to the reference gene *HPRT* and vehicle group. The data represent three independent experiments and are presented as the mean ± SEM. Statistical significance was determined by a two-tailed Student‘s *t*-test. * *p* < 0.05; ** *p* < 0.01; *** *p* < 0.001; ns, not significant.

**Figure A1:**
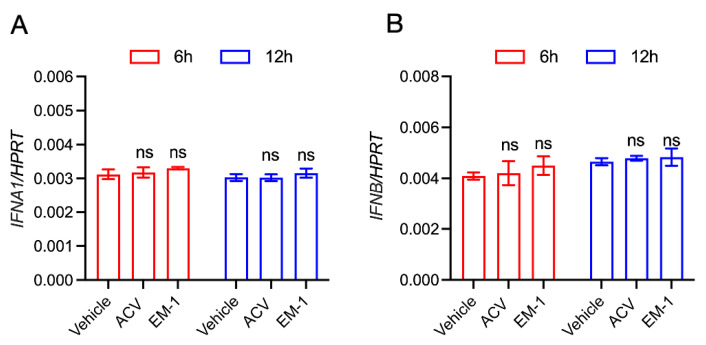
EM-1 showed no significant modulation of type I IFNs. (related to Figure 4). (**A**) Total RNA was extracted and subjected to RT-qPCR for detection of mRNA levels. RT-qPCR analysis of *IFNA1* in U251 cells at 6 h or 12 h with 10 µM EM-1 and 10 µM ACV. The expression levels of *IFNA1* are presented relative to the reference gene *HPRT* and vehicle group. (**B**) RT-qPCR analysis of *IFNB* in U251 cells at 6 h or 12 h with 10 µM EM-1 and 10 µM ACV. The expression levels of *IFNB* are presented relative to the reference gene *HPRT* and vehicle group. The data represent three independent experiments and are presented as the mean ± SEM. Statistical significance was determined by a two-tailed Student‘s *t*-test. ns, not significant.

To elucidate the mechanism underlying the anti-HSV-1 activity of EM-1, U251 cells were treated with 10 μM EM-1 for 6 or 12 h. Total RNA was extracted and subjected to RT-qPCR for detection of mRNA levels. As shown in Figure 4, EM-1 induced significant upregulation of type II interferon and its downstream effector genes *ISG15* and *MX1* in the 12 h group (*p* < 0.001), with no significant modulation of type I IFNs (Appendix A Figure A1). Notably, the expression levels of these genes were consistently higher in the 12 h group compared to the 6 h group, suggesting a time dependent enhancement of antiviral immune responses induced by EM-1.

### 2.5. The Peptide EM-1 Inhibits Viral Load and Reduces Inflammatory Responses in Mice Lungs

**Figure 5:**
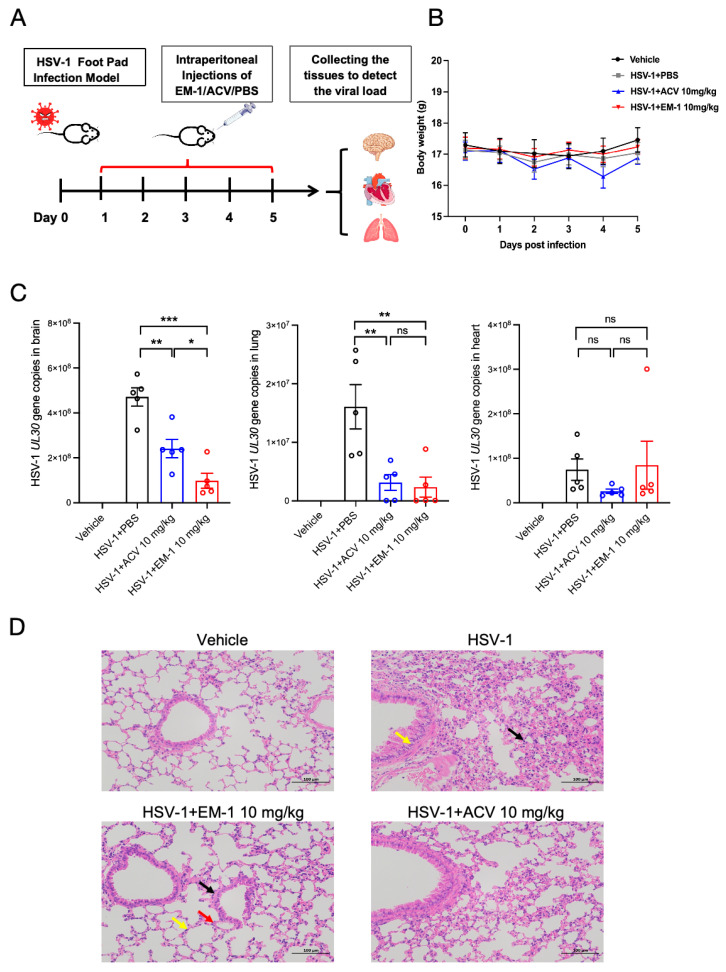
EM-1 significantly inhibits HSV-1 infection in mice via reduction in viral load. (**A**) Schematic representation of the HSV-1-infected mouse model. (**B**) Graphical representation of body weight changes in C57BL/6J mice during five-day consecutive drug administration. Following HSV-1 infection, mice were administered with a daily intraperitoneal injection of either 10 mg/kg EM-1 or 10 mg/kg ACV or an equivalent volume of PBS as a control. The body weights of the mice were monitored over a period of five days post infection (n = 5 mice). (**C**) HSV-1 *UL30* gene copies in whole brain, lung, and heart tissues (n = 5 mice) on day 5 post-infection. Tissue DNA was extracted and subjected to qPCR for detection of *UL30* gene copies. (**D**) H&E staining of pathological sections of mouse lung sections. In the HSV-1 group, the black arrows indicate granulocyte infiltration, while the yellow arrows mark hydropic degeneration of epithelial cells. In the EM-1 treatment group, the black arrows indicate the intrapulmonary bronchi, the yellow arrows mark the alveoli, and the red arrows point to the intrapulmonary connective tissue and blood vessels. The scale bar represents 100 µm. Statistical significance was determined by a two-tailed Student’s *t*-test. * *p* < 0.05; ** *p* < 0.01; *** *p* < 0.001; ns, not significant.

In vivo trials are crucial to assess the therapeutic efficacy of peptides. To determine the anti-HSV-1 of EM-1 efficacy in vivo, we established a mouse infection model via HSV-1 footpad inoculation. Infected mice received daily intraperitoneal administration of EM-1 (10 mg/kg), ACV (10 mg/kg), or PBS vehicle control for five consecutive days. The experimental timeline detailed in Figure 5A, and the monitoring of weight changes throughout the experiment is as shown in Figure 5B. Viral load analysis demonstrated, with the highest burden in the brain (8.67 ± 0.16 log_10_ copies/μL), intermediate levels in the heart (7.78 ± 0.41 log_10_ copies/μL), and lowest detection in the lungs (7.02 ± 0.57 log_10_ copies/μL), as shown in Figure 5C. Notably, compared with the HSV-1 control group, the viral load in brain tissue was significantly reduced in both the EM-1 group (*p* < 0.001) and the ACV treatment group (*p* < 0.01). Moreover, EM-1 achieved a significantly greater reduction in viral load than ACV (*p* < 0.05). In the lung tissue, compared with the HSV-1 control group, viral load was significantly reduced in both the EM-1 and ACV treatment groups (*p* < 0.01), with no significant difference in two treatment groups. In heart tissue, neither the EM-1 nor the ACV treatment group showed a significant difference in viral load compared with the HSV-1 control group. Those results indicate potent suppression of HSV-1 replication in the brain and lungs by EM-1 and ACV.

Furthermore, histopathological evaluation through hematoxylin eosin (H&E) staining demonstrated differential therapeutic efficacy across experimental groups in lung tissues. Compared with the HSV-1 control group, both EM-1 and ACV treatment groups preserved normal alveolar architecture with intact epithelial linings and absence of inflammatory infiltrates in interstitial compartments (Figure 5D).

## 3. Discussion

HSV-1 remains a significant global health challenge, causing diseases ranging from mild mucosal lesions to life-threatening encephalitis and neonatal infections. Current therapeutic treatments are limited by two major challenges: viral latency in sensory ganglia and increasing antiviral resistance. These limitations highlight the urgent need for new therapeutic approaches [25]. Antiviral peptides (AVPs) have emerged as a promising therapeutic alternative due to their unique mechanisms. Their ability to target conserved viral surface structures enables broad spectrum activity against enveloped viruses through membrane fusion inhibition [26]. Furthermore, AVPs exhibit low resistance propensity owing to polypharmacological mechanisms that simultaneously disrupt multiple viral processes. Based on these advantages, this study investigated Asian elephant (*E. maximus*) derived cathelicidin as an anti-HSV-1 candidate. Its native amphipathic α-helical structure makes it an ideal template for designing antiviral peptides. Through template based design, we preserved the native α-helical core to maintain viral glycoprotein interaction domains while implementing strategic cationic residue substitutions (Arg/Lys). These modifications enhanced the net positive charge to promote electrostatic viral membrane adsorption, in accordance with Matsuzaki’s charge threshold principle [27]. Biophysical optimization further improved the therapeutic potential of the modifed peptides. Concurrently, hydrophobic residues (Phe, Leu) were positioned within the hydrophilic face of the α-helix. This design preserves transmembrane permeability while minimizing hydrophobic interactions with cholesterol in host cell membranes. In our study, in vitro cytotoxicity and hemolysis assays confirmed that the optimized peptide EM-1 had significantly improved safety profiles compared to the template peptide EM.

Cathelicidin peptides, evolutionarily conserved components of host defense systems, exhibit multifunctional biological activities, including direct antimicrobial effects and immunoregulatory functions. Their indirect antiviral mechanisms principally involve immune modulation through intracellular signaling cascade activation, establishing systemic antiviral states characterized by elevated interferon (IFN) and pro-inflammatory cytokine production. Recent studies have highlighted the potential of cathelicidins against HSV-1. For example, Hg-CATH and Pb-CATH4 demonstrated significant inhibition of HSV-1 replication in human keratinocytes without direct virucidal effects, suggesting their action likely involves interference with intracellular viral processes or host immunomodulation [28]. WL-1, a truncated 16 amino acid peptide derived from LL-37, exhibits potent anti-HSV-1 activity in vitro and in vivo. WL-1 not only reduced viral loads in epithelial and neuronal cells but also mitigated HSV-1 induced facial palsy in mice, likely by disrupting viral replication and dampening inflammatory responses [29]. These findings collectively underscore the versatility of cathelicidins as anti-HSV-1 agents, with structural features and mechanisms dictating their therapeutic potential. The modified cathelicidin EM-1 fits into this landscape as a novel derivative combining immunostimulatory properties with optimized stability, offering a promising candidate for anti-HSV-1 therapy. In our study, the modified cathelicidin EM-1 demonstrated significant anti-HSV-1 activity in U251 cells, which was associated with the upregulation of interferon-γ and its downstream gene expression. The observed selectivity of EM-1 in inducing IFN-γ (type II IFN) without significant modulation of IFN-α/β (type I IFN) suggests a unique immunomodulatory profile. IFN-γ is primarily produced by activated T cells, especially Th1 subsets and NK cells, whereas type I IFNs are typically secreted by virus-infected cells such as plasmacytoid dendritic cells or innate immune sensors. EM-1 may be preferentially internalized by antigen presenting cells that prime Th1 responses, leading to IFN-γ dominated signaling. It is very important to conduct further experiments using a greater variety of cells. Future studies also should explore EM-1’s comparative efficacy against other cathelicidins and its potential synergy with existing antivirals to address drug resistant HSV-1 strains. The observed upregulating of IFN-γ and downstream ISGs indicate that EM-1 may potentially elicit antiviral activity against other viruses. It is necessary to conduct confirmation of its broad-spectrum antiviral efficacy on other viruses.

In vivo assessment, providing superior clinical relevance compared to in vitro models, was implemented to evaluate EM-1’s therapeutic efficacy. The HSV-1-infected mouse model was established via footpad inoculation. Our results revealed that viral loads are 8.67 ± 0.16, 7.78 ± 0.41, and 7.02 ± 0.57 log_10_ copies/μL in whole brain, heart, and lung tissues, respectively. The brain exhibits the highest viral load compared to the heart and lungs, suggesting a pronounced neurotropism of HSV-1. This observation aligns with the well-documented ability of HSV-1 to establish latent infections in sensory ganglia and subsequently reactivate them, leading to neurological complications such as encephalitis [30]. Compared with the HSV-1 group, EM-1 administration significantly reduced brain tissue viral load to 7.91 ± 0.44 log_10_ copies/μL (*p* < 0.001) and lung tissue viral load to 6.37 ± 0.23 log_10_ copies/μL (*p* < 0.01), demonstrating its potent anti-HSV-1 activity in the brain and lung. Furthermore, the viral load in the brain tissue of the ACV treatment group decreased to 8.36 ± 0.25 log_10_ copies/μL (*p* < 0.01), and the viral load in the lung tissue decreased to 6.01 ± 0.13 log_10_ copies/μL (*p* < 0.01). These results indicate that ACV also exhibits anti-HSV-1 activity in the brain and lungs. Notably, EM-1 demonstrates a significantly better effect than ACV in reducing the viral load in brain tissue (*p* < 0.05), while there is no significant difference in the viral load in lung tissue between the two groups. We also observed that neither the EM-1 nor the ACV treatment group could reduce the viral load in the heart. The limited cardiac impact, while requiring further mechanistic investigation, suggests distinct tissue specific antiviral mechanisms. EM-1 significantly reduced brain viral loads, indicating that it may cross the blood brain barrier or modulate neuroinflammatory responses that facilitate viral clearance. The reduced viral load in the lung following EM-1 treatment may be attributed to the robust innate immune defenses of the lung, including alveolar macrophages, and potentially relates to antiviral mechanisms of EM-1 such as direct virion disruption or immune modulation. Notably, ACV is a standard treatment for HSV-1. Its mechanism depends on viral thymidine kinase [31]. In contrast, EM-1 likely acts through TK independent pathways that may have broader tissue efficacy. Inflammatory activation serves as a fundamental innate immune mechanism against pathogenic invasion or tissue injury. HSV-1 causes tissue damage through both viral cytopathy and dysregulated immune responses [32]. Our results demonstrate that EM-1 treatment mitigated these histopathological aberrations. EM-1 may have the ability to suppress inflammation and potentiate viral clearance while exerting broad anti-inflammatory effects in lung tissue.

Our results identify elephant cathelicidin derived EM-1 as a promising therapeutic peptide, highlighting the value of utilizing host-derived antimicrobial peptides for novel antiviral development and demonstrating its clinical potential. However, comprehensive preclinical validation remains essential for therapeutic advancement.

## 4. Materials and Methods

### 4.1. Cells and Virues

Human glioma cells (U251) were maintained in Dulbecco’s Modified Eagle Medium (DMEM; Gibco, Waltham, MA, USA) supplemented with 10% heat-inactivated fetal bovine serum (FBS; ExCell), 100 U/mL penicillin, and 100 µg/mL streptomycin. Serum inactivation was performed at 56 °C for 30 min prior to medium supplementation. Cell cultures were incubated at 37 °C under 5% CO_2_ atmosphere. The HSV-1 17 + strain utilized in this study was maintained under laboratory preservation conditions.

### 4.2. Peptides Design and Synthesis

The NCBI database (https://www.ncbi.nlm.nih.gov/) was systematically queried using the keywords “*Elephas maximus*” and “Cathelicidin” to identify potential antimicrobial peptide templates. The cathelicidin mature peptide from *E. maximus* was identified and designated as the template peptide EM. Through sequence optimization, four modified peptides EM-1, EM-2, EM-3, and EM-4 were designed. Specifically, the charge of the original EM was modified by substituting neutral/polar residues with arginine (Arg) and lysine (Lys) to enhance cationic charge, thereby improving antimicrobial activity. Arg was prioritized due to its bidentate hydrogen bonding with lipid head groups, which promotes deeper membrane insertion and stronger destabilization. Lys was incorporated to maintain high biocompatibility while contributing to the net positive charge. Hydrophobic residues leucine (Leu), isoleucine (Ile), valine (Val), and phenylalanine (Phe) were inserted or retained in the EM1–4 peptides to achieve 40–60% hydrophobicity, balancing hydrophobicity to maximize antimicrobial activity while minimizing hemolytic toxicity. In addition, the α-helical domain of EM was preserved, as it is essential for pore formation and membrane disruption. The polar/nonpolar residue ratio was maintained at approximately 50% for structural flexibility and biocompatibility [33]. C-terminal amidation was implemented to enhance peptide stability. All synthetic peptides were commercially synthesized by GL Biochem Ltd. (Shanghai, China), with purity exceeding 95% as verified through reversed-phase, high performance liquid chromatography (RP-HPLC) and mass spectrometric analysis (Appendix A Table A1).

### 4.3. Prediction of Physicochemical Properties of Peptides

(1) Physicochemical properties including molecular weight, theoretical isoelectric point, and grand average of hydropathicity (GRAVY) were predicted using the ProtParam tool (https://web.expasy.org/protparam/, 29 December 2022).
(2) Transmembrane helix domains were analyzed through TMHMM (https://services.healthtech.dtu.dk/services/TMHMM-2.0/, 29 December 2022).
(3) Subcellular localization prediction was performed with Cell-PLoc 2.0 (http://www.csbio.sjtu.edu.cn/bioinf/Cell-PLoc-2/, 29 December 2022).

### 4.4. Cytotoxicity Assay

Cell viability was evaluated by using cell counting kit-8 reagent (CCK-8, Biosharp, Shanghai, China) according to the manufacturer’s protocol. Briefly, U251 cells were seeded in 96-well culture plates and cultured for 12 h at 37 °C. The cells were then treated with or without peptides for 24 h. After that, 10 μL of the CCK-8 reaction mixture were added to each well and incubated at 37 °C for 0.5–4 h. The absorbance of each well was measured at 450 nm with a SpectraMax M2e Multi-Mode Microplate Reader (Molecular Devices, USA). The formula for calculating cell viability is as follows:

Cell viability (%) = (average OD of treated sample-average OD of vehicle sample)/(average OD of cell sample-average OD of vehicle sample) × 100%

### 4.5. Hemolysis Analysis

Hemolysis assay was tested with rabbit erythrocytes in a liquid medium. Serial dilutions of the testing peptides were used, and after incubation at 37 °C for 30 min, the cells were centrifuged and the absorbance in the supernatant was measured at 450 nm to determine the hemolysis (%) of peptides. Maximum hemolysis was determined by adding 0.1% Triton X-100 to a sample of cells.

### 4.6. Isolation DNA and RNA for qPCR and RT-qPCR Analyses

U251 cells were seeded into 12-well plates at a density of 2 × 10^5^ cells per well and incubated for 12 h. Subsequently, the cells were co-incubated with HSV-1 at an MOI of 0.1 combined with EM-1 or ACV for 24 h. Following treatment, the total DNA was extracted by using DNA isolation mini kit (Vazyme, Nanjing, China) under the manufacturer’s instructions.

Total RNA was extracted using TRIzol reagent (Invitrogen, Carlsbad, CA, USA). One microgram of RNA was reverse-transcribed into cDNA by using a reverse transcription kit (Vazyme, China). Diluted gDNA and cDNAs were subjected to qPCR analysis using ChamQ Universal SYBR qPCR Master Mix (Vazyme, China) with the following parameters: 95 °C for 30 s, followed by 40 cycles of 95 °C for 5 s and 60 °C for 30 s, followed by melting curve analysis. Primers for qPCR were synthesized by Sangon Biotech (Shanghai, China) (Appendix A Table A2). The qPCR data were analyzed using the 2^−ΔΔCt^ method.

### 4.7. Animal Experiment

Female C57BL/6J mice, aged 6–8 weeks, were purchased from Beijing Vital River Laboratory Animal Technology Co., Ltd. (Beijing, China). After a 7-day acclimatization period, the mice were randomly divided into four groups: vehicle control group, HSV-1 virus control group, HSV-1 + EM-1 (10 mg/kg) treatment group, and HSV-1 + positive drug ACV (10 mg/kg) treatment group. Except for the vehicle control group, mice in the other groups were subplantarly infected with HSV-1 at a dose of 1.0 × 10^6^ PFU per mouse. Drug treatment (EM-1 or ACV) was initiated via intraperitoneal injection 1 h post-infection and administered once daily for 5 consecutive days. The vehicle control and virus control groups received an equivalent volume of phosphate-buffered saline (PBS). Mice were euthanized 24 h after the last administration, and tissue samples were collected. Body weight was measured daily at a fixed time during the treatment period to generate weight change curves.

Tissues were homogenized using a tissue grinder and total DNA was extracted from homogenate supernatants using a DNA isolation mini kit (Vazyme, China) under the manufacturer’s instructions. To establish a standard curve for real-time quantitative PCR (qPCR), the *UL30* gene was used as the target sequence, and a recombinant plasmid containing this gene was constructed. In brief, the *UL30* gene was cloned into the pMD19-T vector and transformed into DH5α. After verification by colony PCR and sequencing, high-purity plasmid DNA was extracted, and its concentration was measured using Nanodrop and converted into copy numbers (copies/μL). Then a 10-fold serial dilution (10^1^–10^8^ copies/μL) was prepared to generate the standard curve. Using the extracted DNA as a template, *UL30* gene copy numbers were quantified in mouse tissues through qPCR with *UL30* specific primers.

Tissues were fixed in 4% paraformaldehyde for 72 h and subsequently processed by Servicebio Biotechnology Co., Ltd. (Wuhan, China) for paraffin embedding, pathological sectioning, and hematoxylin eosin (H&E) staining.

### 4.8. Statistical Analysis

All the data presented were analyzed using GraphPad Prism software (version 8.0; GraphPad Software, Boston, MA, USA). The results are expressed as the mean ± SEM. A two-tailed Student’s *t*-test or a two-way ANOVA with multiple comparison correction was used to analyze the differences among the multiple groups. A *p*-value of < 0.05 was considered to be statistically significant.

## Data Availability

The original contributions presented in this study are included in the article. Further inquiries can be directed to the corresponding author(s).

## Appendix A

**Table A1:** The amino acid sequences and the physicochemical properties of template peptide EM and its modified peptides.

Physicochemical Property	EM	EM-1	EM-2	EM-3	EM-4
Sequence	RPRHVRRIRGLRKFFRKSKEKLKKVGRRVKGFFRDVLRRVPYLPGPRFSYAF-NH_2_	RRIRGLRKFFRKSKEKLKKV-NH_2_	GRRVKGFFRDVLRRVPYLPGPR-NH_2_	RVIRVLKKFFKKIKKILKKV-NH_2_	VRKVKKFFKKVLKKVKKLVRVI-NH_2_
Length	52	20	22	20	22
Charge	+18	+10	+6	+10	+11
Theoretical MW	6459.80	2574.21	2642.15	2513.33	2712.58
Measured MW	6458.72	2573.19	2641.13	2512.31	2711.56
Isoelectric point	12.21	12.19	12.01	12.06	12.07
Molecular formula	C_299_H_488_N_100_O_61_	C_117_H_209_N_41_O_24_	C_121_H_197_N_41_O_26_	C_123_H_222_N_34_O_21_	C_132_H_239_N_37_O_23_
Hydrophilia	Hydrophilic	Hydrophilic	Hydrophilic	Hydrophilic	Hydrophilic
Stability	Unstable	Stable	Unstable	Stable	Stable
Subcellular localization	Cytoplasm	Cytoplasm	Cytoplasm	Cytoplasm	Cytoplasm

**Table A2:** The primer sequences.

Gene	Primer Sequences (5′→3′)
HSV-1 *UL30*	F: TGTTTCGCGTGTGGGACATA
	R: TTGTCCTTCAGGACGGCTTC
*18S rRNA*	F: GTAACCCGTTGAACCCCATT
	R: CCATCCAATCGGTAGTAGCG
*HPRT*	F: GCTATAAATTCTTTGCTGACCTGCTG
	R: AATTACTTTTATGTCCCCTGTTGACTGG
*IFNG*	F: CCAGAGCATCCAAAAGAGTGTG
	R: TCAGCTTTTCGAAGTCATCTCGTT
*MX1*	F: GTTTCCGAAGTGGACATCGCA
	R: CTGCACAGGTTGTTCTCAGC
*ISG15*	F: CGCAGATCACCCAGAAGATCG
	R: TTCGTCGCATTTGTCCACCA
*IFNA1*	F: CTCATACACCAGGTCACGCT
	R: AGTGTAAAGGTGCACATGACG
*IFNB*	F: AGCTGAAGCAGTTCCAGAAG
	R: AGTCTCATTCCAGCCAGTGC
